# Development of a core predictor set of weaning in critically ill patients: a Delphi-based study protocol

**DOI:** 10.3389/fmed.2024.1483011

**Published:** 2024-10-29

**Authors:** Danqiong Wang, Linya He, Yan Chen, Keqi Pan, Meng Wu, Meng Zhou, Weiwen Zhang, Zubing Mei, Guozheng Zhang

**Affiliations:** ^1^Department of Critical Care Medicine, The Quzhou Affiliated Hospital of Wenzhou Medical University, Quzhou People's Hospital, Quzhou, China; ^2^The Second School of Clinical Medicine, Zhejiang Chinese Medical University, Hangzhou, China; ^3^School of Medicine, Shaoxing University, Shaoxing, Zhejiang, China; ^4^Department of Anorectal Surgery, Shuguang Hospital, Shanghai University of Traditional Chinese Medicine, Shanghai, China; ^5^Department of Radiology, The Quzhou Affiliated Hospital of Wenzhou Medical University, Quzhou People's Hospital, Quzhou, China

**Keywords:** core predictor set, weaning, critically ill, intensive care unit, Delphi study

## Abstract

**Introduction:**

Prolonged mechanical ventilation in intensive care units (ICUs) leads to increased morbidity, higher mortality rates, and elevated healthcare costs. Predicting successful weaning from mechanical ventilation with accuracy is essential for optimizing resource use and improving patient outcomes. The International Classification of Functioning, Disability and Health (ICF) framework offers a holistic perspective on health conditions and can be adapted to identify key predictors of weaning readiness. This study aims to develop a Delphi-based core predictor set for weaning in critically ill patients, utilizing the ICF model.

**Methods and analysis:**

The core predictor set development comprises three steps: (1) Literature review and expert consultation to gather weaning predictors, (2) Predictor alignment with ICF categories per established rules, and (3) Three-round Delphi survey with a multidisciplinary team. A systematic review across major databases will be conducted to identify predictors related to weaning predictors in critically ill adults from cohort studies, trials, and reviews. Predictors will then be categorized within ICF domains. A multidisciplinary expert panel will evaluate the relevance of each predictor using a 9-point Likert scale to achieve consensus.

**Discussion:**

This study will contribute to the development of a standardized, evidence-based predictor set for weaning readiness in critically ill patients. Using the ICF framework, this study aims to encompass the complex factors that influence weaning, thereby enabling personalized care plans and improving weaning outcomes. The Delphi methodology guarantees a thorough, iterative process for building consensus by integrating diverse clinical perspectives.

**Conclusion:**

The proposed Delphi-based study protocol aims to establish a core set of predictors for weaning in the ICU setting, guided by the ICF model. Successful implementation of this predictor set could enhance decision-making around weaning trials, reduce unnecessary ventilation days, and ultimately improve patient outcomes and healthcare efficiency. Future validation and implementation studies will be essential to confirm the utility and generalizability of this predictor set in clinical practice.

## Introduction

Prolonged mechanical ventilation within intensive care units (ICUs) poses significant challenges, exacerbating patient morbidity, elevating mortality rates, and inflating healthcare expenditures (1–3). Prolonged mechanical ventilation is generally defined as requiring ventilatory support for more than 14–21 days, although the exact duration may vary depending on the clinical context and specific patient factors (4). The intricate task of predicting successful weaning from ventilatory support with precision is paramount to optimize resource allocation and ensure improved clinical outcomes for critically ill patients (5, 6). Current methods of evaluating and predicting the outcome of weaning include various physiological tests, such as spontaneous breathing trials (SBTs), rapid shallow breathing index (RSBI), and arterial blood gas analysis, as well as clinical assessments like the weaning index and patient-specific comorbidities (7, 8). However, these methods often lack standardization and may not comprehensively account for all relevant factors affecting weaning success (26, 27).

The lack of a standardized, evidence-based predictor set for assessing weaning readiness currently contributes to variability in weaning practices and inconsistencies in patient management strategies. The International Classification of Functioning, Disability and Health (ICF) has assumed a pivotal role in advancing the clinical understanding and management of complex conditions, particularly in predicting weaning success among critically ill patients (9, 10). By adopting a bio-psychosocial perspective, the ICF goes beyond traditional biomedical models to encompass a broad spectrum of factors influencing patient outcomes, including body functions, activity limitations, participation restrictions, environmental factors, and personal characteristics (11–13). Several studies have demonstrated the necessity of using the ICF and Delphi methods in this field, highlighting their ability to integrate diverse perspectives and achieve consensus on key predictors (14, 15). For instance, the ICF has been used to develop comprehensive rehabilitation plans and to identify functional outcomes that are meaningful to patients and clinicians alike (16, 17). Similarly, the Delphi method has been successfully applied in critical care settings to establish core outcome sets and improve the consistency of clinical practice (18, 19).

In the context of weaning from mechanical ventilation, the ICF framework facilitates a comprehensive assessment of predictors, such as respiratory muscle strength, cognitive function, and social support, which are crucial for tailored weaning strategies. This approach has led to research focused on identifying and validating multidimensional predictors, which improves the accuracy of weaning prediction models and supports clinical decision-making. Consequently, the ICF's application in this arena not only contributes to optimized weaning protocols and reduced ICU stays but also fosters a patient-centered approach that considers the broader impact of critical illness on an individual's functioning and overall wellbeing. Thus, the ICF has catalyzed progress in personalized medicine within intensive care, underlining the importance of an integrated approach to care in improving weaning outcomes for critically ill patients.

The literature on predicting weaning success in critically ill patients reveals a substantial inconsistency in reported results (8, 20–29), stemming from the heterogeneity of studied populations, varying definitions of successful weaning, and the multitude of predictive factors considered. Studies often employ different assessment tools and statistical methodologies, leading to conflicting evidence on the efficacy of specific predictors such as respiratory mechanics, ventilator parameters, and clinical indices. This diversity in approaches highlights the complexity of weaning prediction and underscores the need for standardized outcome measures and a unified predictor set to enhance comparability and reliability across investigations. The absence of a consistent core outcome set has hindered the synthesis of evidence and the formulation of definitive guidelines, necessitating further research aimed at achieving consensus and refining our understanding of optimal weaning predictors in the ICU setting.

This study aims to address this critical gap by developing a core predictor set of weaning in critically ill patients, grounded in the comprehensive framework of ICF. Prolonged mechanical ventilation is associated with augmented morbidity, mortality, and escalated healthcare expenses; hence, the objective of the study is to enhance weaning decision-making, optimize resources, and ameliorate patient outcomes. The methodology employed in this protocol follows a meticulous three-stage process. Initially, a systematic review of the extant literature is executed across major databases to collate a comprehensive catalog of potential weaning predictors. Secondly, a modified Delphi study engaging a multidisciplinary panel of stakeholders will be initiated. Through iterative rounds of Delphi surveys, experts from disciplines such as intensive care, respiratory therapy, physiotherapy, nursing, and other allied health professions rate the predictors' relevance to weaning success. Lastly, consensus will be sought for the development and validation of this predictor set, defined by statistical agreement criteria, ensuring the robustness of the predictor set and clinical applicability. The successful implementation of our proposed predictor set could, therefore, be a pivotal step toward optimizing ICU resource utilization, enhancing patient recovery trajectories, and advancing the quality of critical care worldwide.

## Methods and analysis

### Study design

Our study employs a rigorous, multi-stage approach to develop a core predictor set for weaning in critically ill patients, underpinned by the principles of the Delphi methodology (30, 31) and the holistic framework of ICF (32, 33). This study encompasses the systematic review, ICF linking, and the Delphi consensus process. This study has been approved by the ethics committee of the local hospital (Approval Number: 2024-063).

### Systematic review

We commence with an updated comprehensive systematic review of the literature to identify all relevant studies reporting weaning predictors in adult critical ill patients based on the previously published systematic reviews (34, 35). Two independent reviewers will screen titles, abstracts, and full-text articles for eligibility, with disagreements resolved by a third reviewer. Extracted data will encompass predictor variables, assessment methods, and predictive models.

### ICF linking

Post-review, identified predictors will be systematically linked to the ICF domains following established guidelines. This step ensures a comprehensive categorization of predictors into body functions, body structures, activities and participation, environmental factors and personal factors, reflecting the complex interplay of elements influencing weaning. The reported summarized predictor domains based on the ICF are presented in Table 1.

**Table 1:** Reported predictor domains of weaning in critically ill patients based on the International Classification of Functioning, Disability and Health (ICF).

**ICF domain**	**Subdomain**	**Predictor examples**
Body functions	Respiratory function	Respiratory rate, tidal volume, rapid shallow breathing index (RSBI), minute ventilation, PaO_2_/FiO_2_ ratio
	Cardiovascular function	Heart rate, blood pressure, cardiac output, central venous pressure (CVP), systemic vascular resistance (SVR)
	Neuromuscular function	Diaphragm excursion, maximal inspiratory pressure (MIP), quadriceps strength, peripheral nerve stimulation tests
	Cognitive function	Sedation level, Glasgow Coma Scale (GCS), Richmond Agitation-Sedation Scale (RASS)
Body structures	Lung structure	Chest radiograph abnormalities, CT scan findings, lung compliance
	Airway integrity	Secretion amount, cuff leak test, airway edema
Activities and participation	Breathing control	Spontaneous breathing trial (SBT) duration, breathing pattern stability
	Mobilization	Bedside mobility, sitting balance, standing, ambulation
Environmental factors	Equipment support	Ventilator modes (PSV, SIMV), PEEP levels, FiO_2_ settings
	Care setting	ICU staff expertise, nurse-to-patient ratio, noise levels
	Social support	Family presence, emotional support, rehabilitation team involvement
Personal factors	Age	Age groups, pediatric vs. adult vs. geriatric
	Comorbidities	Chronic lung disease, obesity, renal insufficiency
	Psychological factors	Anxiety, depression, motivation, understanding and compliance

ICF, the International Classification of Functioning, Disability and Health.

### Delphi process

The Delphi process in our study will adopt a digital approach, leveraging Google forms as the platform for administering the Delphi survey (36). We chose Google Forms over Microsoft Excel for several reasons: the user-friendly interface, facilitation of remote and asynchronous participation, automatic data compilation, and real-time updates, which are crucial for engaging a geographically dispersed multidisciplinary panel of experts. This choice facilitates remote and asynchronous participation, enabling engagement from a geographically dispersed multidisciplinary panel of experts, including intensivists, respiratory therapists, physiotherapists, nurses, and other allied health professionals experienced in weaning management.

Participants will be presented with a list of predictors that have been systematically linked to the ICF domains post-literature review. Each predictor will be evaluated using a 9-point Likert scale (37, 38), as recommended by the Grading of Recommendations Assessment, Development and Evaluation (GRADE) working group and the Core Outcome Measures in Effectiveness Trials (COMET) initiative (39–41). The 9-point Likert scale is designed to capture the perceived importance of each predictor in assessing weaning success. Participants will rate each predictor as follows:

- Scores 7–9: critically important. A score in this range indicates that the predictor is considered essential for inclusion in the core set due to its high relevance to weaning success.
- Scores 4–6: important but not critical. These scores suggest the predictor is relevant but not indispensable for weaning success and may not need to be part of the final core set.
- Scores 1–3: low importance. Predictors receiving a score in this range are considered less relevant and may not warrant inclusion in the core set.
- “Unable to score”: this option is provided if participants feel they lack the expertise or knowledge to appropriately evaluate a particular predictor.

This scale allows for nuanced scoring and helps distinguish between predictors that are absolutely critical, moderately important, or of low significance. Importantly, this process helps build consensus while allowing for differences in expert opinion.

To ensure confidentiality when sending forms to different participants, we implemented several measures. Google Forms can be configured to collect responses anonymously, ensuring that participants' identities are not linked to their responses. We used secure links to share the forms, ensuring that only invited participants can access the surveys. Additionally, Google Forms uses end-to-end encryption to protect data during transmission and storage, and responses are stored securely on Google's servers, which comply with strict data protection standards. These measures collectively ensure the privacy and security of participant data throughout the Delphi process.

Google forms will be configured to automatically compile responses while maintaining anonymity, ensuring participants' confidentiality and promoting candid feedback. The Delphi rounds will span over a 3-week period each, with two reminder emails scheduled to prompt participants who have not yet completed the survey. This timeframe strikes a balance between allowing ample reflection and maintaining momentum in the process of building consensus. After each round, results will be synthesized and fed back to participants, highlighting areas of consensus and disagreement. The Delphi process will iterate through at least three rounds or until a stable consensus is reached, defined by predetermined criteria.

### Sample size

Determining the optimal sample size for Delphi studies is challenging due to the iterative and consensus-driven nature of the method. However, Core Outcome Set-STAndards for Development (COS-STAD) recommendations do not provide a specific formula for calculating the number of respondents (42). Nonetheless, previous study suggests a minimum of seven respondents per stakeholder group to allow for meaningful consensus (43). Acknowledging potential attrition and to ensure robust representation across all stakeholder categories, we aim to invite a minimum of 20 participants per group. We considered an attrition rate of ~30% based on previous Delphi studies in similar contexts (44). This calculation was based on the assumption that a 30% attrition rate would leave us with around 14 participants per group, which aligns with the recommendation of having at least seven respondents per stakeholder group to allow for meaningful consensus (45). Therefore, inviting 20 participants per group provides a buffer to maintain sufficient participation even after accounting for potential dropouts.

Participants will be selected to ensure geographical, professional, and experiential diversity, ensuring global representation and a comprehensive perspective on weaning predictors. Inclusion of stakeholders from high-income, middle-income, and low-income countries will capture variations in resource availability and cultural practices. Special attention will be given to ensuring balanced participation from all professional backgrounds to reflect the multidisciplinary nature of weaning decisions in ICUs.

To ensure global representation, participants will be recruited from a minimum of five continents, covering high-, middle-, and low-income countries. Specifically, for each stakeholder group, we plan to have 10 participants (50%) from high-income countries, six participants (30%) from middle-income countries, and four participants (20%) from low-income countries. This distribution is justified by the need to include perspectives from countries with advanced healthcare systems and resources, which are crucial for developing a comprehensive predictor set (high-income countries, 50%). Middle-income countries (30%) represent a significant portion of the global population and have varying levels of resource availability, providing a balanced view. Including participants from low-income countries (20%) ensures that the predictor set is applicable in resource-limited settings and reflects the unique challenges faced in these regions.

### Data collection and management

In the context of the Delphi process, data collection and management are critical to ensuring a transparent, confidential, and structured consensus-building exercise. Below are the detailed procedures adopted for managing the Delphi survey data.

#### Survey administration and response collection

Google Forms, chosen for its accessibility and ease of use, will serve as the platform for administering the Delphi survey. The survey will be piloted among a small subset of experts before the main study to test clarity, functionality, and estimated completion time. All questions will be formatted to maintain anonymity and prevent identification of individual responses.

#### Consistency and anonymity

To preserve participant anonymity and ensure unbiased responses, no personally identifiable information will be collected within the survey forms. Participants will receive a unique identifier known only to the research team for tracking response rates across rounds but without connecting responses to individuals.

#### Data entry and cleaning

Upon submission, Google forms will automatically compile responses into a Google sheets document, minimizing manual data entry errors. A designated research team member will review the responses for completeness and consistency. In case of unclear or ambiguous responses, participants will be contacted for clarification without compromising their anonymity.

#### Data synthesis and feedback

After each Delphi round, the research team will analyze the data, calculating means, medians, interquartile ranges, and percentages of agreement for each predictor. This synthesis will be compiled into a summary report, excluding any identifiers, which will then be circulated to participants before the next round. The report will highlight areas of consensus, ongoing disagreements, and any new comments or suggestions from participants.

#### Management of iterative rounds

To maintain the momentum and ensure continuous engagement, reminders will be sent out electronically at predefined intervals to participants who have not completed the survey within the allocated time frame. Adjustments to the Delphi survey, such as removal of predictors reaching consensus or introduction of new ones suggested by participants, will be made between rounds as necessary.

#### Data security

Stringent measures will be taken to protect the confidentiality and security of the data collected. Access to the Google sheets containing Delphi responses will be restricted to authorized members of the research team.

### Phase 1: systematic review

The systematic review constitutes the first phase of our study and serves as the foundation for identifying potential weaning predictors. The review protocol will be registered in PROSPERO, an international prospective register of systematic reviews, to enhance transparency and reduce duplication of research efforts. The systematic review will also adhere to the Preferred Reporting Items for Systematic Reviews and Meta-Analyses (PRISMA) guidelines to ensure completeness and transparency in the reporting of the methodology, data collection, and results. The PRISMA checklist will be followed throughout the process, from the selection of studies to the presentation of findings, thus aligning our work with internationally recognized standards.

#### Search strategy

We will conduct a comprehensive search across multiple databases including PubMed, Embase, and Cochrane Library. Our search strategy will include a combination of keywords and Medical Subject Headings (MeSH) terms specifically designed to capture all relevant literature. The keywords will include “weaning,” “mechanical ventilation,” “ICU,” “predictors,” and “critical illness.” MeSH terms will complement these keywords to ensure a broad and inclusive retrieval of applicable studies.

The search will span from the inception of each database to the present to ensure complete coverage of the literature. Specific filters, such as language restrictions (English only) and study types (clinical trials, cohort studies, and systematic reviews), will be applied to streamline the search process. Additionally, gray literature sources such as conference proceedings, dissertations, and clinical trial registries will be systematically reviewed using tailored search terms related to the study focus on weaning predictors in critically ill patients, to minimize publication bias.

#### Eligibility criteria

Studies will be included if they involve adult patients (≥18 years) in the ICU undergoing weaning from mechanical ventilation, report predictors of weaning success or failure, and are published in English. Weaning success will be defined according to standard criteria, including sustained spontaneous breathing without reintubation within a specified time frame. Exclusion criteria will include studies focusing solely on pediatric populations, non-ventilated patients, or those not reporting original data on weaning predictors (Table 2).

**Table 2:** Inclusion and exclusion criteria for the systematic review.

**Category**	**Inclusion criteria**	**Exclusion criteria**
Study design	Randomized controlled trials (RCTs), observational studies (prospective or retrospective cohort, case-control), and systematic reviews/meta-analyses	Case reports, case series, editorials, letters, comments, animal studies, *in vitro* studies, and studies not written in English
Population	Adult patients (≥18 years old) admitted to intensive care units (ICUs) who are on mechanical ventilation and undergoing weaning or considered for weaning	Pediatric populations, non-ICU patients, patients not on mechanical ventilation, and studies focused on specific diseases unrelated to weaning
Intervention/exposure	Studies that evaluate predictors or factors associated with successful or unsuccessful weaning from mechanical ventilation	Studies that solely focus on interventions without assessing predictors, or those evaluating weaning protocols without predictor analysis
Outcome	Primary outcome: successful weaning, defined as sustained spontaneous breathing without reintubation within a specified timeframe after extubation	Studies not reporting clear weaning outcomes, focusing solely on ventilator settings or management without outcome assessment
Publication	Studies published from inception to the date of the search, including gray literature such as conference abstracts, dissertations, and clinical trial registries	Non-peer-reviewed publications, outdated studies not reflecting current practice, and duplicates

#### Screening and data extraction

Two independent reviewers will conduct the screening of titles, abstracts, and full texts. The eligibility criteria will include: (1) studies involving adult ICU patients undergoing weaning from mechanical ventilation; (2) studies reporting original data on predictors of weaning success or failure; (3) publications in English. Exclusion criteria will encompass pediatric studies, reviews, and non-original research articles. In the event of disagreements, a third, senior reviewer will arbitrate to reach a consensus.

**Figure 1:**
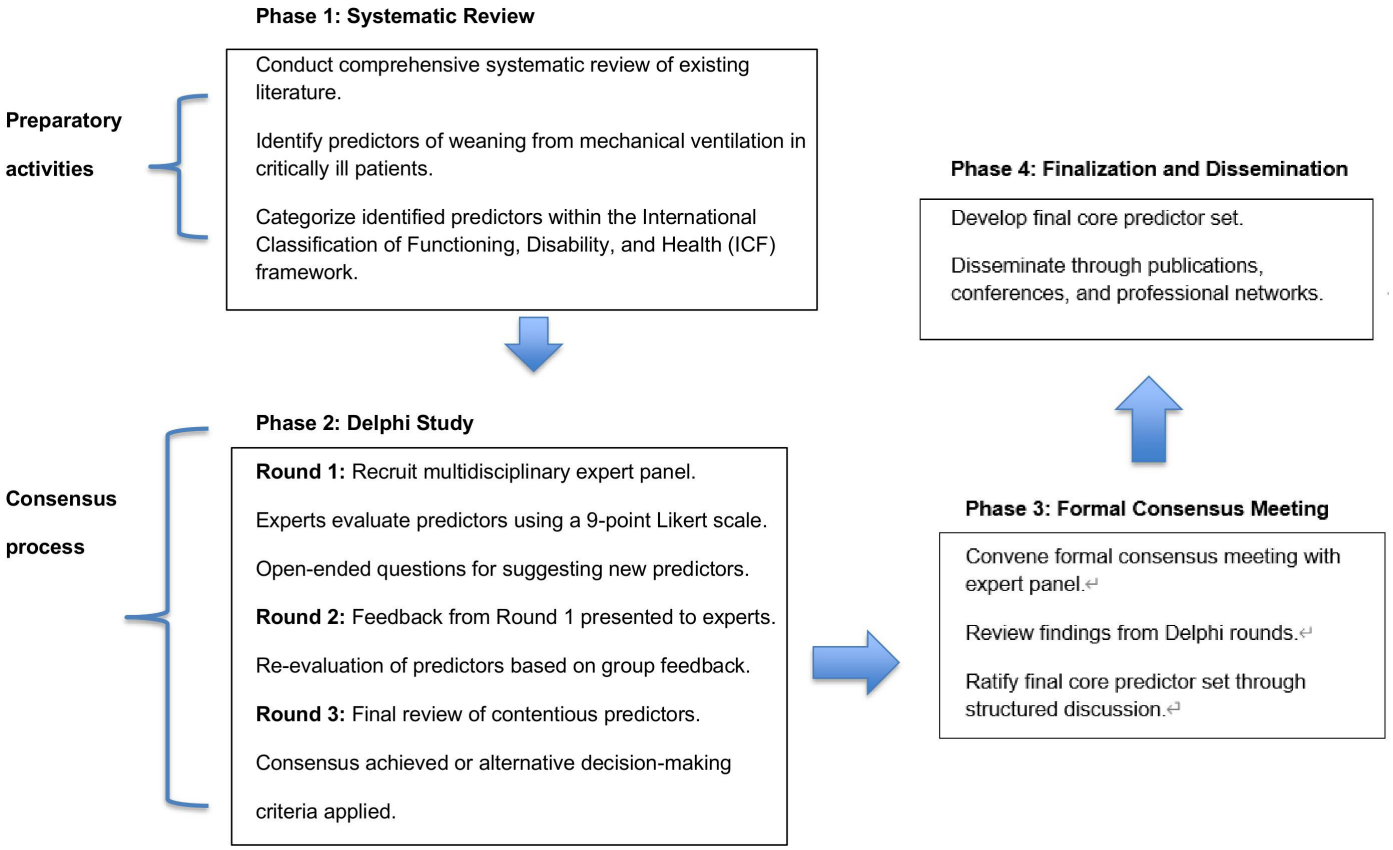
Flowchart for the development of a core predictor set of weaning in critically ill patients.

A detailed flowchart is provided to illustrate the screening process from the initial number of records identified through databases and additional sources to the final selection of included studies (Figure 1).

Data extraction will be systematically performed using a standardized data extraction form specifically designed for this study. This form will capture key data items, including:

1. Study characteristics: study design (e.g., randomized controlled trial, cohort study), year of publication, country, and setting (e.g., ICU type, patient population).
2. Population characteristics: number of patients, age, sex, severity of illness, and relevant clinical features (e.g., comorbidities).
3. Predictor variables: all predictors related to weaning success or failure, including physiological variables, biomarkers, and clinical assessments.
4. Assessment methods: how predictors were measured (e.g., type of ventilator settings, physiological monitoring tools).
5. Predictive models: model types (e.g., logistic regression, machine learning), performance metrics (e.g., sensitivity, specificity, area under the curve).
6. Outcome measures: weaning success, weaning failure, and any relevant secondary outcomes such as mortality or length of stay.
7. Statistical analyses: statistical techniques employed to identify predictors and assess the performance of predictive models.

For missing data, we will first contact the study authors for clarification or additional data if possible. If missing data cannot be obtained, we will describe the nature and extent of the missing data and apply appropriate imputation methods where applicable (e.g., multiple imputation) or exclude studies from specific analyses based on the degree of missing data.

To ensure consistent data extraction across reviewers, we will conduct a pilot test of the data extraction form on a subset of included studies. The results of the pilot will be discussed and any discrepancies will be addressed to refine the form. Additionally, two independent reviewers will perform the extraction for each study. Any disagreements will be resolved through discussion, with a third reviewer consulted if necessary. The quality of evidence will be assessed using the Cochrane Risk of Bias tool for randomized controlled trials and the Newcastle-Ottawa Scale for cohort and case-control studies.

### Phase 2: international online Delphi study

The process of developing core predictor set of weaning in critically ill patients is summarized in Table 3 and described in details as follows.

**Table 3:** Developing core predictor set process of weaning in critically ill patients based on the COMET-COS-STAD.

**Domain**	**Methodology**	**Application in the proposed study**
Scope	Setting	ICU settings where weaning trials are conducted
	Condition	Weaning from mechanical ventilation in critically ill patients
	Population	Adult critically ill patients (18+) undergoing weaning
	Intervention	Weaning protocols using a core set of predictors
	Users	Intensivists and healthcare professionals involved in weaning
Stakeholders	Healthcare professionals	Intensivists, respiratory therapists, and critical care nurses
	Patients	Critically ill patients undergoing weaning from mechanical ventilation
	Others	Methodology experts in Delphi surveys and evidence synthesis
	Consensus	Initial list	Literature review and expert panel input
A priori scoring processing and consensus definition		Delphi rounds methodology as described in the study protocol
A priori inclusion and exclusion criteria		Set criteria based on the literature review and input from the expert panel
Avoid ambiguity of language used in the list of outcomes		Ensure clear and clinically relevant terminology in predictor selection

#### Delphi participants: stakeholder selection and recruitment

Our stakeholder selection and recruitment process aims to ensure a balance of clinical specialties, geographic representation, and professional experiences to enrich the consensus-building process. Key stakeholder groups will include intensivists, respiratory therapists, physiotherapists, critical care nurses, clinical researchers, and bioethicists. The selection process will prioritize individuals with a minimum of 5 years of experience in ICU settings, direct involvement in weaning processes, and a publication record or recognized expertise in the field. Additionally, we will strive for gender and age diversity to mitigate potential biases.

To ensure global representation, participants will be recruited from a minimum of five continents, covering high-, middle-, and low-income countries. This approach acknowledges the influence of resource availability and cultural practices on weaning strategies and aims to develop a predictor set applicable across different healthcare systems.

Stakeholder recruitment will be executed through a multi-pronged approach, meticulously designed to ensure a panel of experts with profound knowledge and extensive clinical experience. We seek professionals from specialties such as critical care medicine, respiratory therapy, intensive care nursing, or related disciplines, holding senior positions to guarantee a breadth and depth of perspectives crucial for comprehensive insight. A minimum of 5 years' hands-on experience in managing critically ill patients, specifically involving ventilator management and weaning processes, is required to ensure that participants are well-versed in the practical intricacies of weaning decision-making. Preference will be given to those who have published research or contributed to guideline development pertinent to weaning in the past 5 years, aligning expertise with contemporary research trends and clinical guidelines. To promote global applicability, we aim for geographical and institutional diversity among participants, encompassing large academic hospitals, community hospitals, and research centers, reflecting differing resource contexts and real-world practices in weaning management. A commitment to active participation throughout all rounds of the Delphi survey is essential, with a willingness to contribute specialist knowledge, engage in open dialogue, and respect the views of peers to facilitate consensus building.

#### Delphi round 1

The first round of the Delphi survey aims to establish an initial assessment of the importance of each predictor variable identified from the systematic review and ICF linking phase. Participants will receive an electronic survey link via email, directing them to a customized Google form. Each predictor will be rated on a 9-point Likert scale, accompanied by a brief description and supporting evidence from the literature where available.

Participants will also have the opportunity to provide open-ended comments, suggest additional predictors, or justify their ratings. The round will remain open for 3 weeks, with two reminder emails scheduled for non-responsive participants. Upon closure, responses will be analyzed, and predictors receiving a median score of 7–9 and an interquartile range ≤ 2 will be considered for retention. Others will be re-evaluated or excluded based on the feedback and rationale provided.

#### Delphi round 2

The second round focuses on refining the predictor set based on the feedback and consensus achieved in Round 1. Participants will receive a summary report outlining the results of the previous round, highlighting areas of agreement and disagreement. Predictors reaching consensus will be confirmed, while those with ongoing controversy will be re-presented for reevaluation, alongside any newly suggested predictors from the open-ended responses.

This round will also employ the 9-point Likert scale, emphasizing the need for reconsideration in light of group feedback. Participants will be encouraged to review their previous scores and adjust as necessary. Again, the survey will be open for 3 weeks, with reminders sent accordingly.

#### Delphi round 3

The final round aims to confirm the core predictor set through achieving definitive consensus. Only predictors that did not achieve consensus in previous rounds will be presented, along with a synthesis of evolving opinions and any remaining disagreements. Participants will be asked for a final evaluation, considering the collective wisdom accumulated throughout the process.

If consensus is not reached after three rounds, a decision will be made based on predefined criteria, such as stability of opinions, level of disagreement, and the clinical relevance of predictors. The final Core Predictor Set will be disseminated through a report detailing the entire Delphi process, justifications for included and excluded predictors, and recommendations for future research and implementation.

### Phase 3: Development of the final core predictor set

#### Formal consensus meeting

Following the Delphi study, a formal consensus meeting will be convened to finalize the core predictor set. This in-person or virtual meeting will invite key contributors from the Delphi rounds, along with additional invited experts to ensure a comprehensive representation of viewpoints. The objective is to review the outcomes of the Delphi process, discuss any remaining disagreements, and ratify the final set of predictors.

During the meeting, facilitators will present a summary of the Delphi findings, including the levels of agreement reached, points of controversy, and the reasoning behind participants' decisions. Open discussion sessions will encourage debate and exploration of alternative perspectives, with the goal of reaching a unanimous decision on the predictor set. If needed, voting mechanisms may be employed to resolve persistent disagreements.

#### Expert panel meeting

An expert panel, consisting of international leaders in critical care medicine, respiratory therapy, physiotherapy, and patient advocacy, will convene to review and endorse the final core predictor set. The panel will assess the clinical relevance, feasibility, and potential impact of the predictor set in clinical practice, research, and policy. Their endorsement will add credibility and ensure the set aligns with best clinical practices worldwide.

The expert panel meeting will also address the practical implications of implementing the core predictor set, discussing issues such as standardization of assessments, training requirements, and potential barriers to adoption. Recommendations for integrating the predictor set into clinical pathways and electronic health records will be formulated.

### Statistical analysis

Once all data is collected, we will employ a combination of descriptive and inferential statistical methods to analyze the data from the Delphi rounds. For descriptive statistics, we will calculate means, medians, standard deviations, and interquartile ranges to summarize the data. For inferential statistics, we will use non-parametric tests such as the Mann–Whitney *U*-test and the Kruskal–Wallis test to compare responses across different stakeholder groups and rounds. Additionally, we will use logistic regression models to identify factors associated with consensus on core predictors.

### Patient and public involvement

Recognizing the importance of patient-centered care, we will incorporate patient and public involvement (PPI) in the final stages of the project. A PPI advisory group, comprising former ICU patients and family members, will be established. They will review the proposed core predictor set and provide insights from the patient perspective, ensuring the set considers patient experiences, preferences, and values.

Feedback from the PPI group will inform the development of patient-centered materials, such as educational resources and consent processes, which can facilitate understanding and engagement during the weaning process. This involvement aligns with the increasing emphasis on shared decision-making and enhances the ethical and societal value of the project.

### Ethics and dissemination

Ethical approval will be sought from the relevant institutional review boards or ethics committees for all stages of the study involving human participants, including the Delphi study and PPI activities. Confidentiality and anonymity of participants will be maintained throughout, with informed consent obtained from all involved parties.

Dissemination plans encompass publication in peer-reviewed scientific journals, presentations at national and international conferences, and engagement with clinical societies and guideline developers. The Core Predictor Set will be made freely available on a dedicated website, accompanied by a user guide detailing its application, interpretation, and potential benefits. Additionally, social media and press releases will be utilized to maximize visibility and encourage uptake by healthcare providers, researchers, and policymakers.

To ensure the practical utility of the Core Predictor Set, a structured plan for monitoring and evaluating its long-term effects will be implemented. This plan will involve:

- A follow-up study to assess changes in weaning outcomes (e.g., success rates, duration of mechanical ventilation, reintubation rates) after the adoption of the Core Predictor Set in clinical practice.
- Collaboration with clinical registries to track and analyze key weaning-related metrics over time.
- Periodic surveys and feedback mechanisms targeting healthcare providers and institutions that have adopted the Core Predictor Set, focusing on its impact on decision-making and patient outcomes.
- Engagement with guideline developers to periodically review the evidence supporting the Core Predictor Set, with updates or modifications made based on new research or clinical insights.

Long-term monitoring and evaluation of the Core Predictor Set's impact on weaning outcomes and clinical practice will be integrated into future research agendas. This ongoing evaluation will help refine the Core Predictor Set, ensuring its relevance and effectiveness in diverse clinical settings.

## Discussion

The proposed study protocol, aimed at developing a core predictor set for weaning in critically ill patients using the ICF framework, holds significant promise for advancing our understanding and management of weaning processes in intensive care settings. The Delphi-based methodology, underpinned by a rigorous systematic review and expert consensus, ensures a comprehensive and evidence-driven approach to identifying key predictors of weaning success.

A pivotal finding of this study is the structured identification and prioritization of weaning predictors across various domains of the ICF model. By integrating predictors from body functions, structures, activities and participation, environmental factors, and personal factors, our study encapsulates the multifaceted nature of weaning readiness. This holistic perspective not only recognizes the biological and physiological aspects but also emphasizes the role of psychological, social, and environmental influences, thus aligning with the biopsychosocial model of healthcare.

Clinically, the adoption of this core predictor set could lead to transformative improvements in patient care. Personalized weaning strategies, informed by a standardized predictor set, can be tailored to individual patient profiles, enhancing the precision of weaning decisions. This may result in reduced durations of mechanical ventilation, minimizing complications associated with prolonged ventilation, such as ventilator-associated pneumonia, muscle weakness, and cognitive impairment. Moreover, the reduction in unnecessary ventilation days translates into healthcare cost savings and optimized resource utilization, benefitting both patients and healthcare systems.

Our study builds upon and extends the findings of previous research in the field of weaning predictors. A systematic review by Trudzinski et al. (35) comprehensively identified a wide range of risk factors for prolonged mechanical ventilation and weaning failure, including patient demographics, comorbidities, and physiological parameters. However, this review noted significant variability in the predictors reported across different studies, underscoring the need for standardized criteria. In contrast, single-predictor studies, such as the systematic review and meta-analysis by de Meirelles Almeida et al. (46), focus on specific factors like diastolic dysfunction and provide valuable insights into their impact on weaning outcomes. While these studies offer detailed analyses of individual predictors, they are limited in scope and do not address the broader array of factors that may influence weaning success. Our Delphi-based study protocol addresses these limitations by achieving consensus among a diverse panel of international experts, thereby developing a more standardized and comprehensive predictor set that integrates both physiological and patient-centered factors. This approach enhances the external validity and clinical applicability of the predictor set, making it a valuable tool for improving weaning outcomes in intensive care units globally.

One of the strengths of this study lies in its multidisciplinary approach. Engaging intensivists, respiratory therapists, physiotherapists, nurses, and other allied health professionals ensures that diverse clinical insights inform the predictor set, enhancing its practical applicability and acceptability. The Delphi methodology, with its iterative consensus-building process, guarantees that the predictor set reflects a broad consensus among experts, fostering trust and promoting its widespread adoption.

However, this study has several limitations that should be acknowledged. Despite our efforts to recruit a diverse panel of experts from various geographic regions and professional backgrounds, there is a potential for selection bias in participant recruitment. While we aim to include participants from high-, middle-, and low-income countries, the majority of participants may still come from high-income countries due to differences in access to resources and research networks. This could limit the generalizability of the findings to resource-limited settings. Additionally, the inherent subjectivity of the Delphi method poses another limitation. The Delphi technique relies on expert opinion and consensus, which can be influenced by individual biases and varying levels of expertise. Although we have established rigorous criteria for participant selection and will use statistical methods to analyze the data, the subjective nature of the process remains a challenge. The iterative nature of the Delphi rounds may also lead to participant fatigue, potentially affecting their engagement and the quality of their responses over time. Furthermore, the study faces logistical challenges, such as coordinating across multiple time zones and languages, which can introduce delays and communication barriers. Ensuring consistent and timely participation from all stakeholders throughout the study period will require careful planning and ongoing support. Finally, the generalizability of the core predictor set may be limited by the specific context of the ICU settings included in the study. While we aim to include a wide range of institutions, the unique characteristics of individual ICUs, such as patient populations and local practices, may influence the relevance and applicability of the predictor set. Future validation studies will be necessary to assess the performance of the core predictor set in diverse clinical settings.

Looking ahead, the successful development of this predictor set paves the way for future validation and implementation studies. Validation in diverse clinical settings will be crucial to ascertain its generalizability and predictive accuracy. Implementation strategies should focus on integrating the predictor set into clinical decision support systems, developing user-friendly interfaces, and providing training to healthcare providers to ensure its effective use. Longitudinal studies tracking patient outcomes post-implementation will provide empirical evidence of its impact on clinical practice and patient-centered outcomes.

In conclusion, this Delphi-based study protocol represents a significant stride toward standardizing weaning practices and improving patient outcomes. By harnessing the power of a multidisciplinary expert panel and the comprehensive ICF framework, it outlines a roadmap for developing a core predictor set that is evidence-based, clinically relevant, and globally applicable. The successful implementation of this predictor set promises to revolutionize weaning management in ICUs, optimizing patient care, reducing healthcare costs, and ultimately contributing to better patient outcomes and quality of life. Future study endeavors should concentrate more on validating and disseminating this predictor set, ensuring its translation into routine clinical practice, and realizing its full potential to transform critical care medicine.
